# Self-Assembly and Enzyme Responsiveness of Amphiphilic Linear-Dendritic Block Copolymers Based on Poly(*N*-vinylpyrrolidone) and Dendritic Phenylalanyl-lysine Dipeptides

**DOI:** 10.3390/polym11101625

**Published:** 2019-10-08

**Authors:** Junwu Wei, Feng Lin, Dan You, Yangyang Qian, Yujia Wang, Yunmei Bi

**Affiliations:** College of Chemistry and Chemical Engineering, Yunnan Normal University, Kunming 650500, China; 15559728219@163.com (J.W.); flin18582316106@163.com (F.L.); youdan526@foxmail.com (D.Y.); 1017767643@pecxy.com (Y.Q.); wangyugaga@yeah.net (Y.W.)

**Keywords:** linear-dendritic block copolymers, dendritic phenylalanyl-lysine dipeptides, poly(*N*-vinylpyrrolidone), micelles, enzyme responsiveness, disassembly

## Abstract

In this study, we present the synthesis, self-assembly, and enzyme responsive nature of a unique class of well-defined amphiphilic linear-dendritic block copolymers (PNVP-*b*-*dendr*(Phe-Lys)_n_, n = 1–3) based on linear poly(*N*-vinylpyrrolidone) (PNVP) and dendritic phenylalanyl-lysine (Phe-Lys) dipeptides. The copolymers were prepared via a combination ofreversible addition-fragmentation chain transfer (RAFT)/xanthates (MADIX) polymerization of *N*-vinylpyrrolidone and stepwise peptide chemistry. The results of fluorescence spectroscopy, ^1^H NMR analyses, transmission electron microscopy (TEM), and particle size analysis demonstrated that the copolymers self-assemble in aqueous solution into micellar nanocontainers that can disassemble and release encapsulated anticancer drug doxorubicin or hydrophobic dye Nile red by trigger of a serine protease trypsin under physiological conditions. The disassembly of the formed micelles and release rates of the drug or dye can be adjusted by changing the generation of dendrons in PNVP-*b*-*dendr*(Phe-Lys)_n_. Furthermore, the cytocompatibility of the copolymers have been confirmed using human lung epithelial cells (BEAS-2B) and human liver cancer cells (SMMC-7721). Due to the fact of their enzyme responsive properties and good biocompatibility, the copolymers may have potential applicability in smart controlled release systems capable of site-specific response.

## 1. Introduction

Enzyme responsive polymers are highly promising carriers for targeted drug delivery because of their excellent biocompatibility and high degree of selectivity [1,2,3,4]. Some enzymes are frequently overexpressed in inflamed or tumor tissues [5,6]. By incorporating enzyme labile linkages, macromolecular carriers are able to be engineered to release the loaded drug on demand, which holds great potential in reducing the exposure to healthy cells and tissues [7]. According to the expression level of the enzyme in a specific region, the release rate of the drug can be controlled by interactive adjustment [8,9]. In addition, enzyme responsive polymers have shown high potential for long-term cycling and targeted therapy and diagnosis of disease in vivo [10]. However, although some enzyme responsive linear-linear block copolymers have been investigated, until recently, only a few studies related to enzyme responsive linear-dendritic block copolymers (LDBCs) have been reported [11,12].

Linear-dendritic block copolymers (LDBCs) combine the easy synthesis, availability, and processability of linear polymeric chains with the regular and well-defined structure of dendrons, which have an exact number of peripheral groups that can be modified for the introduction of functionality in a controlled manner [13,14,15]. Amphiphilic LDBCs self-assemble in aqueous solution into micellar nanocontainers that can encapsulate and release hydrophobic payloads such as drug molecules. In drug delivery systems, compared to other vehicles consisting of only linear block copolymers or dendrimers, the benefit of using LDBCs nanocontainers as carriers is that they combine the high functionality imparted by the dendrimer with the high loading capacity of the block copolymer, which not only increases the stability of self-assembly in aqueous solutions but also can be customized to optimize their load capacity and release rates for different payloads [16].

Amino acid-based polymers have received considerable attention for their pharmaceutical and other medical applications because of their excellent biocompatibility and good biodegradability [17,18]. *L*-lysine has been extensively utilized as remarkable building blocks to construct the dendrons of LDBCs for biomedical applications, such as drug delivery [19,20], tissue engineering [21], and gene therapy [22] in the past decades. However, the studies on enzyme responsive LDBCs containing amino acids moiety still remain largely unexplored. On the other hand, some small peptide motifs composed of several different amino acids can be cleaved by tumor-associated extracellular proteases (such as matrix metalloproteinases) or lysosomal cysteine proteases (such as cathepsins and trypsin) [23,24]. Trypsin, previously known as a digestive enzyme produced by pancreatic acinar cells, has been confirmed to be expressed in a variety of cancers and thought to be involved in tumor proliferation, invasion, and metastasis [25,26,27,28], whereas trypsin can cleave solely C-terminal to lysine or arginine [29]. 

Because of good antifouling property, low toxicity, and biocompatibility, poly(ethylene glycol) (PEG) is often used as a hydrophilic material, is modified to the surface of nanocarriers to increase their stability, resist protein adsorption in vivo, and prolong the circulation of nanocarriers in the body [30,31]. Consequently, most of enzyme-responsive LDBCs used PEG as a hydrophilic linear link to hydrophobic branches which can be hydrolyzed by lipase or amidase [32,33,34]. However, a large number of studies and clinical results have shown that PEG materials are slowly showing many shortcomings. The PEG-modified nanocarriers tend to enrich in liver and spleen tissues [35]. Multiple intravenous injections of PEG-modified nanocarriers promote accelerated blood clearance (ABC) and produce PEG antibodies in vivo [36,37]. These deficiencies of PEG ultimately affect the dose and therapeutic effect of PEG nanomedicine, or even cause toxic side effects. Thus, it is necessary to explore possible PEG alternatives and develope branches with better biocompatibility [38,39].

Little attention has been paid to poly(*N*-vinylpyrrolidone) (PNVP)-containing linear-dendritic block copolymers (LDBCs). Poly(*N*-vinylpyrrolidone) (PNVP), as a highly hydrophilic polymer, was used as a plasma solubilizer as early as World War II [40]. And PNVP-modified nanoparticles can also increase the stability of nanoparticles and can effectively resist protein adsorption in vivo. Moreover, PNVP-containing amphiphilic block copolymers have unique advantages over PEG-containing amphiphilic block copolymers in drug delivery applications, because the polymeric micelles with PNVP outer shell have not aggregation problems during lyophilization [41,42,43].

In this study, we have designed and synthesized a unique class of well-defined enzyme responsive amphiphilic linear-dendritic block copolymers (PNVP-*b*-*dendr*(Phe-Lys)_n_, n = 1–3) on the basis of linear poly(*N*-vinylpyrrolidone) (PNVP) and dendritic dipeptides phenylalanyl-lysine (Phe-Lys). Their aggregation behaviors in aqueous solution were fully characterized by fluorescence spectroscopy, ^1^H NMR analyses, transmission electron microscopy (TEM), and particle size analysis. The ability of the micelles to encapsulate small hydrophobic guests and to release them in the presence of a serine protease trypsin was investigated using Nile red dye and anticancer drug doxorubicin. The in vitro cytotoxicity of PNVP-*b*-*dendr*(Phe-Lys)_n_ (n = 1–3) was also evaluated for potential biomedical application. To the best of our knowledge, this is the first report on synthesis and enzyme responsive properties of linear-dendritic block copolymers (LDBCs) containing PNVP and dendritic phenylalanyl-lysine (Phe-Lys) dipeptides.

## 2. Experimental

### 2.1. Materials

*N*-vinylpyrrolidone (NVP, 98%, Aldrich, St. Louis, MO, USA), was distilled twice under reduced pressure prior to use. α,α-Azobisisobutyronitrile (AIBN) (98%, Sinopharm Chemical Reagent, Shanghai, China) was purified by recrystallizing from methanol two times. The [1-(*O*-Ethylxanthyl)ethyl]benzene(CTA) [44] and tert-butyl (2-acrylamide ethylene) carbamate [45] were made according to the literature. (Fmoc)Phe–OH, (Fmoc)Lys(Fmoc)–OH, 1-hydroxybenzo-triazole, *N*,*N*-dicycloethylcarbodiimide, 4-methyl piperidine and tris(2-carboxyethyl)phosphine (TCEP) were purchased from Sinopharm Chemical Reagent (Shanghai, China). Nile red, doxorubicin (DOX), and trypsin are purchased from Sigma–Aldrich, Shanghai, China. Dialysis bags (MWCO = 3500) were obtained from Gene Star Co. (Shanghai, China). Other reagents (analytical or chromatographic grade) were obtained from Sinopharm Chemical Reagent (Shanghai, China).

### 2.2. Characterizations

The ^1^H NMR spectra were obtained on a Bruker DRX-500 (Rheinstetten, Germany) spectrometer in CDCl_3_, DMSO-d_6_ or D_2_O. Tetramethylsilicone (TMS) was used as internal standards. Molecular weights and molecular weight distributions were measured by gel permeation chromatography (GPC; Waters, Milford, CT, USA) with a Waters 2690D separations module and a Waters 2414 refractive index detector (R). Styragel HR1 and HR4 columns were used at 40 °C with poly(methyl methacrylate) (PMMA) as standards and DMF (included 0.05 M LiBr) as a mobile phase at a flow rate of 0.3 mL/min.

### 2.3. Synthesis of PNVP-b-dendr(Phe-Lys)_n_ (n = 1–3)

#### 2.3.1. Synthesis of PNVP 

The PNVP was synthesized according to the previously published literature [44] in 86.1% yield. ^1^H NMR in CDCl_3_, *δ*(ppm): 7.28–7.12 (m, ArH), 4.63–4.67 (br, OCH_2_), 4.56–4.37 (m, PhCH), 3.90–3.74 (m, NCH), 3.48–3.20 (m, NCH_2_), 2.38–2.19 (m, COCH_2_), 2.07–1.99 (m, CH_2_), 1.71–1.59 (m, CH_2_), 1.43 (s, CH_3_), 1.26 (s, CH_3_).

#### 2.3.2. Synthesis of PNVP–NHBoc

The PNVP (7.2g, *M*_n,th_ = 29020 g/mol) , n-butylamine (3.40 mL, 0.42 mol), and tri-*n*-butylphosphine (0.64 mL) were dissolved in 40 mL CH_2_Cl_2_ solution. Tris(2-carboxyethyl)phosphine (TCEP) (0.172 g, 0.6 mmol) and tert-butyl (2-acrylamide ethylene)carbamate (0.28 g, 0.66 mmol) were added under a nitrogen atmosphere. Then stirred for 6 h, the reaction mixture was poured into cold ether to precipitate the product. The precipitate was redissolved in water, dialyzed, freeze-dried, affording PNVP–NHBoc as a white solid in 78.6% yield (5.66 g). ^1^H NMR in CDCl_3_, *δ*(ppm): 7.28–7.13 (m, ArH), 5.37–5.34 (br, NH), 4.57–4.37 (m, PhCH), 3.91–3.74 (m, NCH), 3.49–3.21 (m, NCH_2_ and SCH_2_), 2.38–2.21 (m, COCH_2_), 2.07–2.00 (m, CH_2_), 1.71–1.59 (m, CH_2_), 1.43 (s, CH_3_).

#### 2.3.3. Synthesis of PNVP–NH_2_

The PNVP–NHBoc (5.6 g, 0.2 mmol) was dissolved in 60 mL of TFA. After stirring for 4 h at room temperature, the solution of pH was adjusted to 9 with 5 N NaOH aqueous solution. The resulting solution was dialyzed and subsequently freeze-dried, affording PNVP–NH_2_ as a white solid in 91.2% yield (5.1 g). ^1^H NMR in CDCl_3_, δ(ppm): 7.28–7.12 (m, ArH), 4.56–4.39 (m, PhCH), 3.92–3.73 (m, NCH), 3.48–3.20 (m, NCH_2_ and SCH_2_), 2.38–2.20 (m, COCH_2_), 2.06–1.99 (m, CH_2_), 1.72–1.59 (m, CH_2_), 1.43 (s, CH_3_).

#### 2.3.4. Synthesis of PNVP-*b*-Phe-NHFmoc 

The PNVP–NH_2_ (2.1 g, 0.1 mmol), 1-hydroxybenzo triazole (13.51 mg, 0.1 mmol), and (Fmoc)Phe–OH (0.12 g, 0.3 mmol) were dissolved in 10 mL of DMF. Then *N*,*N*-dicycloethylcarbodiimide (0.06 mL, 0.4 mmol) was added to the solution and stirred for 24 h at room temperature. The reaction mixture was poured into cold ether to precipitate the product. The precipitate was redissolved in water, dialyzed, freeze-dried, affording PNVP-*b*-Phe-NHFmoc as a white solid in 91.5% yield (1.96 g).^1^H NMR in DMSO-d_6_, δ (ppm): 7.96–7.13 (m, ArH), 6.83–6.75 (br, NH), 6.29 (s, NH), 4.56–4.43 (m, PhCH), 4.30 (q, J = 10 Hz, α-CH(Phe)), 4.17 (t, J = 7.5 Hz, CH(Fmoc)), 4.11 (q, J = 10.5 Hz, CH_2_(Fmoc)), 3.95–3.47 (m, NCH), 3.30–2.85 (m, SCH_2_, NCH_2_ and *β*-CH_2_(Phe)), 2.40–1.99 (m, COCH_2_), 1.98–1.75 (m, CH_2_), 1.74–1.42 (m, CH_2_), 1.32 (s, CH_3_).

#### 2.3.5. Synthesis of PNVP-*b*-Phe-NH_2_

The PNVP-*b*-Phe-NHFmoc (1.95 g, 0.093 mmol) was dissolved in 10 mL of mixed solution of 4-methyl piperidine and DMF (V/V = 1:4). After stirring for 2 h at room temperature, the reaction mixture was poured into cold ether to precipitate the product. The precipitate was redissolved in water, dialyzed, freeze-dried, affording PNVP-*b*-Phe-NH_2_ as a white solid in 94.3% yield (1.84 g). ^1^H NMR in DMSO-d_6_, *δ*(ppm): 7.26–7.15 (m, ArH), 6.83–6.75 (br, NH), 4.52–4.45 (m, PhCH), 4.24 (q, J = 10 Hz, *α*-CH(Phe)), 3.95–3.47 (m, NCH), 3.31–2.86 (m, SCH_2_, NCH_2_ and *β*-CH_2_(Phe)), 2.40–1.99 (m, COCH_2_), 1.98–1.75 (m, CH_2_), 1.74–1.42 (m, CH_2_), 1.32 (s, CH_3_).

#### 2.3.6. Synthesis of PNVP-*b*-dendr(Phe-Lys)

The PNVP-*b*-Phe-NH_2_ (1.8 g, 0.086 mmol), 1-hydroxybenzo triazole (11.56 mg, 0.086 mmol), and (Fmoc)Lys(Fmoc)–OH (100 mg, 0.26 mmol) were dissolved in 10 mL of DMF. Then *N*,*N*-dicycloethylcarbodiimide (0.052 mL, 0.34 mmol) was added to the solution and stirred for 24 h at room temperature. The reaction mixture was poured into cold ether to precipitate the product. The precipitate was redissolved in water, dialyzed, and freeze-dried, affording PNVP-*b*-dendr(Phe-Lys) as a white solid in 87.5% yield (1.58 g).

#### 2.3.7. Synthesis of PNVP-*b*-dendr(Phe-Lys)_2_

Repeat the previous experimental steps on the basis of PNVP-*b*-*dendr*(Phe-Lys) to obtain PNVP-*b*-*dendr*(Phe-Lys)_2_.

#### 2.3.8. Synthesis of PNVP-*b*-dendr(Phe-Lys)_3_

Repeat the previous experimental steps on the basis of PNVP-*b*-*dendr*(Phe-Lys)_2_ to obtain PNVP-*b*-*dendr*(Phe-Lys)_3_. 

### 2.4. Critical Micelle Concentration (CMC) Measurement

Critical Micelle Concentration (CMC) were determined with a Hitich F-4500 luminescence spectrophotometer (Hitachi, Tokyo, Japan) using Nile red as a fluorescent probe [46]. Nile red stock solution (45µL, 0.88 mg/ml in ethanol) were added into 100 mL phosphate buffer solution (pH 7.4), and mixed to give a final concentration of 1.25 µM. A 1.0 mg/mL solution of each polymer (PNVP-*b*-*dendr*(Phe-Lys)_n_ (n = 1–3) was prepared in diluent and sonicated for 15 min. This solution was repeatedly diluted by a factor of 2 with diluent. The solutions containing Nile red were kept at room temperature for 24 h before measurements. The fluorescence emission intensity was scanned for solution of each concentration. Maximum emission intensity was plotted versus polymer concentration in order to determine the CMC. All measurements were repeated three times.

### 2.5. Monitoring Trypsin-Induced Disassembly of the Nile Red-Loaded Micelles

Monitoring trypsin-induced disassembly of the Nile red-loaded micelles was performed using a Hitich F-4500 luminescence spectrophotometer (Hitachi, Tokyo, Japan). The entire experiment was carried out in a 37 °C water bath. The PNVP-*b*-*dendr*(Phe-Lys)_n_ (n = 2, 3) was dissolved in PBS buffer (pH 7.4) to give a concentration of 1.0 mg/mL solution. The solution was sonicated for 15 minutes and then filtered through a 0.45 μm nylon syringe filter. This solution (2.0mL) was transferred to a quartz cuvette and 1.44 μL of Nile red stock solution (0.88 mg/mL in ethanol) was added to give a final concentration of 2.0 μM. A fluorescence emission scan was performed (t = 0) and then 10 μL of trypsin stock solution (16 U/μL in PBS buffer pH 7.4 at 37 °C) was added to give a final concentration of 80 U/mL. Repeating fluorescence scans were performed every 1 h for 48 h.

### 2.6. Particle Sizing and Transmission Electron Microscope (TEM) Measurements

The sizing of the micelles was determined by a ZataPALS particle size analyzer (Brookhaven Instruments Corp, New York, USA). A drop of micellar solution (1 mg/mL, or Nile red loaded micelles, or dealt with 80 U/mL of trypsin stock solution) was placed on a FORMVAR-coated copper grid and dried in air. The TEM images of the micelles were obtained using a JEM-2100 transmission electron microscope (JEOL, Akishima, Tokyo, Japan) at an acceleration voltage of 200 kV.

### 2.7. Loading and Enzyme-Triggered Release of Doxorubicin (DOX) into/from the LDBCs Micelles 

The DOX-loaded micelles were prepared according to the literature [47]. Drug loading (DL) and encapsulation efficiency (EE) were calculated using the following equations: DL (w/w) = (amount of loaded drug)/(amount of polymer), EE (w %) = (actual amount of loaded drug)/(amount of added drug). In vitro release from the DOX-loaded micelles (1 mg/mL) was carried out using dialysis bags in PBS buffer (pH = 7.4) at 37 °C. The enzyme-triggered cargo release was achieved by adding the trypsin (80 U/mL) to the DOX-loaded solution. The amount of DOX released was determined by using fluorescence (Hitich F-4500) measurement (excitation at 480 nm).

### 2.8. In Vitro Cytotoxicity Evaluation 

In vitro cytotoxicity of PNVP-*b*-*dendr*(Phe-Lys)_n_ (n = 2, 3) against human lung epithelial cells (BEAS-2B) and human liver cancer cells (SMMC-7721) was evaluated using the colorimetric MTS assay [48]. Briefly, BEAS-2B cells were seeded into a 96-well plate at a density of 5 × 10^3^ cells per well in 100 μL of Dulbecco’s modified Eagle’s medium (DMEM; Sigma), supplemented with 10% fetal bovine serum (FBS), and incubated in a humidified atmosphere (37 °C, 5% CO_2_) for 24 h. After 24 h, the polymer solutions (100 μL in normal saline) at different concentrations were added to each well. The media were removed after 48 h of incubation, and then a mixture of 20 μL of 3-(4, 5-dimethylthiazol-2-yl)-5(3-carboxymethoxyphenyl)-2-(4-sulfopheny)-2H-tetrazolium (MTS) and 100 μL fresh medium was added to each well. After being incubated for further 4 h at 37 °C, then the absorbance values were measured at 492 nm using a microplate reader (Multiskan FC, Thermo, Waltham, MA, USA).

## 3. Results and Discussion

### 3.1. Synthesis of Amphiphilic LDBCs

**Scheme 1:**
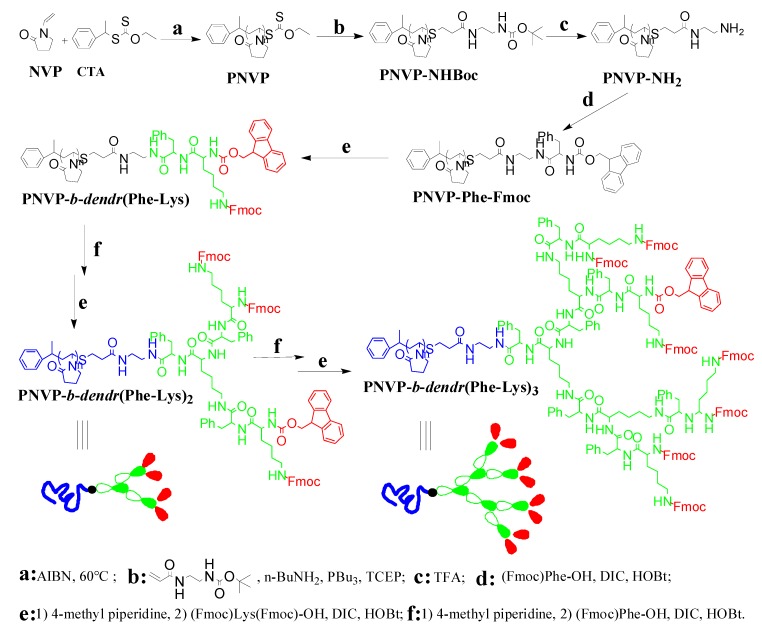
Synthesis of amphiphilic linear-dendritic copolymers.

The synthetic route of three generations of linear–dendritic block copolymers PNVP-*b*-*dendr*(Phe-Lys)_n_ (n = 1–3) was shown in Scheme 1. Firstly, well-defined PNVP with an O-ethylxanthyl end-group was synthesized by reversible addition-fragmentation chain transfer (RAFT)/xanthates (MADIX) polymerization of NVP with a [1-(*O*-ethylxanthyl)ethyl]benzene as a RAFT/MADIX agent. The *O*-ethylxanthyl end-group of PNVP subsequently was reduced via aminolysis, and the resulting thiol was trapped with a Michael acceptor, tert-butyl (2-acrylamide ethylene) carbamate in situ to yield a BocNH-terminated PNVP (PNVP–NHBoc). Then, the protecting Boc group from amino group of PNVP–NHBoc was removed with trifluoroacetic acid to afford amino-terminated PNVP (PNVP-NH_2_). The PNVP–NH_2_ was used as the polymeric supporter, and then PNVP-*b*-*dendr*(Phe-Lys)_n_ (n = 1–3) were synthesized via stepwise peptide chemistry. Each synthesis step was monitored by a ninhydrin test, ^1^H NMR spectra and GPC.

**Figure 1:**
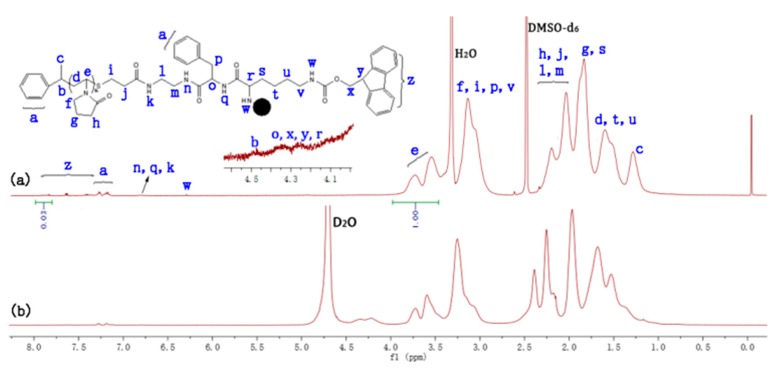
^1^H NMR spectra of PNVP-*b*-*dendr*(Phe-Lys) in DMSO-d_6_ (**a**) and D_2_O (**b**).

The ^1^H NMR spectra of PNVP-*b*-*dendr*(Phe-Lys)_n_ (n = 1–3) were fully consistent with the proposed chemical structures (Figure 1a, Figures S1a and S2a). For example, the proton characteristic signals of the PNVP repeat-units in the ^1^H NMR spectra of PNVP-*b*-*dendr*(Phe-Lys) (Figure 1a) can be expressed in the peaks marked with letters from d to h, d at 1.74–1.42 ppm (m, CH_2_), e at 3.98–3.46 ppm (m, NCH), f at 3.31–2.85 ppm (m, NCH_2_), g at 1.99–1.75 ppm (m, CH_2_), h at 2.40–1.99 ppm (m, COCH_2_), and the resonance signals of dendron protons of Fmoc group and phenyl group at 7.95–7.13 ppm, *α*-methine group of Lys, *α*-methine group of Phe, methine group and methine group of Fmoc group at 4.43–4.16 ppm. 

**Figure 2:**
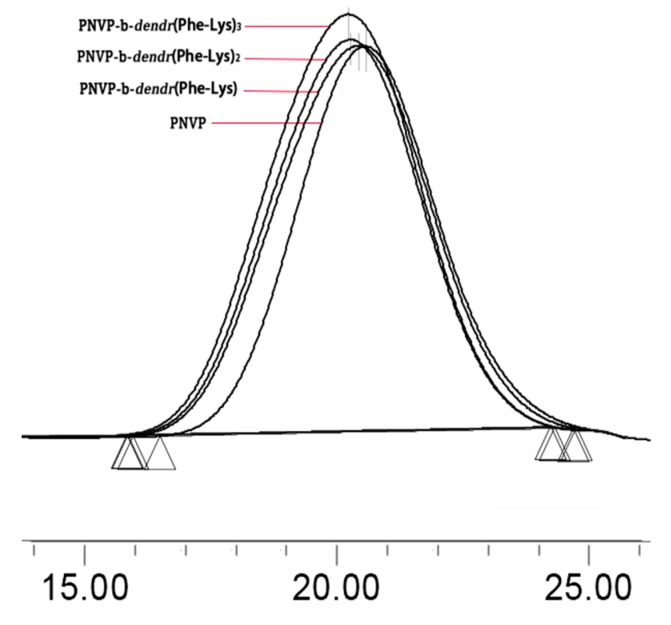
GPC traces of polymers.

The GPC analysis of the copolymers revealed monomodal peaks with narrow molecular weight distributions (*M*_w_*/M*_n_ < 1.35; Figure 2, Table 1). The molecular weights *M*_n,GPC_ of the copolymers increased in the order of PNVP < PNVP-*b*-*dendr*(Phe-Lys) < PNVP-*b*-*dendr*(Phe-Lys)_2_ < PNVP-*b*-*dendr*(Phe-Lys)_3_. The *M*_n,NMR_ values of the LDBCs were determined by comparing the integration ratio of Ar protons of Fmoc in dendritic blocks at 7.28–7.95 ppm (z) with CHN protons of the PNVP backbone at 3.45–4.00 ppm (e). The *M*_n,NMR_ values of LDBCs increased with increasing the generation of the dendritic dipeptides, indicating three generations of copolymers were synthesized.

### 3.2. Self-Assembly of Amphiphilic LDBCs

The amphiphilic PNVP-*b*-*dendr*(Phe-Lys)_n_ were expected to be able to self-assemble in aqueous solution to form micelles with a hydrophobic dendritic Phe-Lys with peripheral Fmoc groups’ cores surrounded by a hydrophilic PNVP shell. To test this, the self-assembled micelles with core–shell structures of PNVP-*b*-*dendr*(Phe-Lys)_n_ were first examined by comparing the ^1^H NMR spectra of the LDBCs in DMSO-d_6_ with in D_2_O (Figure 1, Figures S1 and S2). All protons of the LDBCs can clearly be seen in ^1^H NMR spectra in DMSO-d_6_, because DMSO-d_6_ is a good solvent for the amphiphilic LDBCs with both types of blocks. However, in the ^1^H NMR spectra of PNVP-*b*-*dendr*(Phe-Lys)_n_ in D_2_O, the peaks of the Fmoc group disappeared at 7.28–7.95 ppm and the benzene proton peaks at 7.12–7.30 ppm (Figure 1b, Figures S1b and S2b) originating from Phe are highly suppressed, indicating that the mobility of Fmoc and Phe-Lys groups was limited by the formation of core–shell micellar structures with hydrophobic inner cores and hydrophilic outer shells. The CMC of the LDBCs was determined by encapsulating Nile red as a hydrophobic fluorescent probe. As shown in Figures S3 and S4, the CMC of PNVP-*b*-*dendr*(Phe-Lys)_2_ and PNVP-*b*-*dendr*(Phe-Lys)_3_ were 0.071 and 0.056 mg/mL, respectively, which depends on the hydrophobicity of the dendritic block. When the hydrophobicity of the dendritic block increased, the CMC values of the LDBCs decreased dramatically. It should be noted that the first-generation LDBC, PNVP-*b*-*dendr*(Phe-Lys) does not form a valid envelope for Nile red due to its fewer hydrophobic Ph and Fmoc groups. 

**Figure 3:**
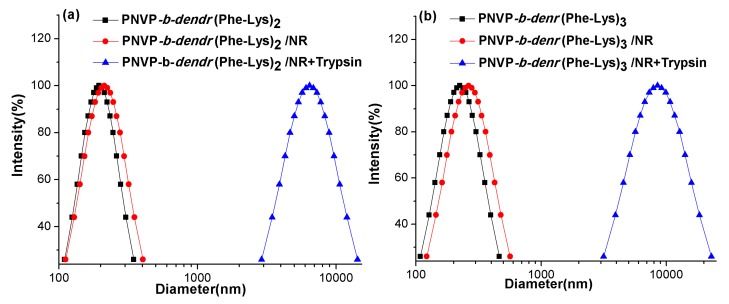
The particle size in PNVP-*b*-*dendr*(Phe-Lys)_2_ (**a**) and PNVP-*b*-*dendr*(Phe-Lys)_3_ (**b**) before and after Nile red was loaded, and the particle size of the micelle solution after the incubation with trypsin.

**Figure 4:**
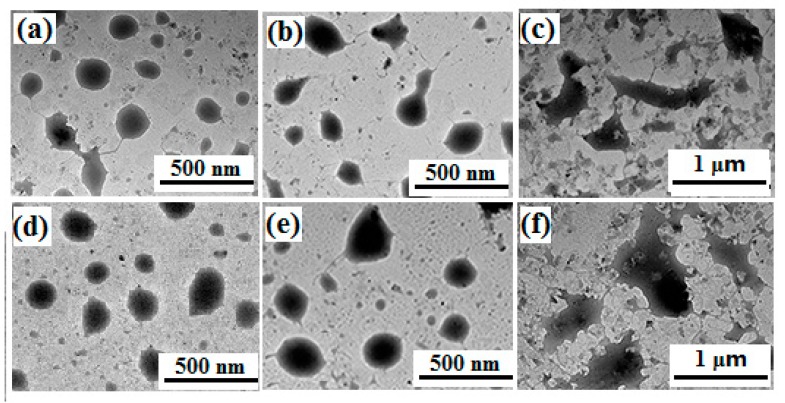
TEM images of the micelles formed by PNVP-*b*-*dendr*(Phe-Lys)_2_ before (**a**) and after (**b**) loaded with Nile red and loaded with Nile red after the incubation with trypsin (**c**), PNVP-*b*-*dendr*(Phe-Lys)_3_, without Nile red (**d**), loaded with Nile red (**e**), and loaded with Nile red after the incubation with trypsin (**f**).

The micellar diameters were determined using particle sizing measurements. The result showed that PNVP-*b*-*dendr*(Phe-Lys)_2_ and PNVP-*b*-*dendr*(Phe-Lys)_3_ formed micelles with sizes of about 195 nm and 224 nm, and a slight increase in the diameter of the micelle after Nile red was loaded (Figure 3). This may arise from the micelles swelling after the inclusion of hydrophobic molecules. The formation of the spherical structures of LDBC micelles can be observed by TEM and the sizes were in agreement with particle sizing measurements (Figure 4). As expected, both particle sizing measurements and TEM show that PNVP-*b*-*dendr*(Phe-Lys)_3_ had a larger micelle diameter compared with PNVP-*b*-*dendr*(Phe-Lys)_2_.

### 3.3. Enzyme-Triggered Disassembly of the LDBC Micelles

**Figure 5:**
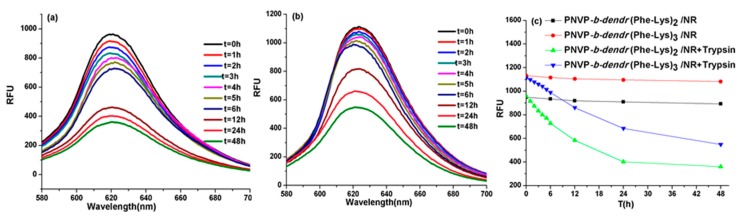
Fluorescence spectra of Nile red in PNVP-*b*-*dendr*(Phe-Lys)_2_ (**a**) and PNVP-*b*-*dendr*(Phe-Lys)_3_ (**b**) micelles after the incubation with trypsin, and effect of adding trypsin on the release rate of Nile Red (**c**).

**Figure 6:**
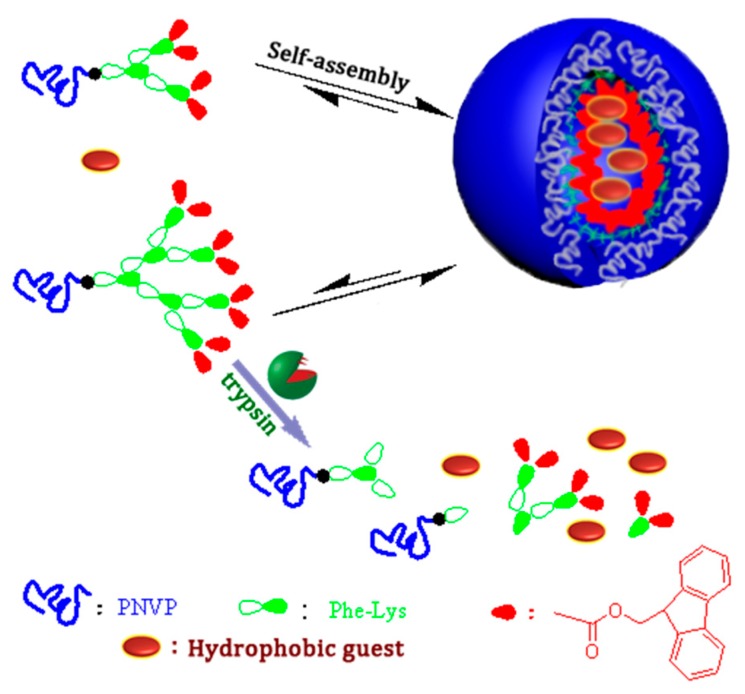
Schematic diagram of self-assembly and enzymatic hydrolysis of PNVP-*b*-*dendr*(Phe-Lys)_n_ (n = 2, 3) with the same hydrophilic block.

The dendritic blocks of the LDBCs consist of dipeptide Phe-Lys which renders them a suitable substrate for trypsin. It is reasonable to expect that the disassembly of LBDC micelles due to the enzymatic degradation of the dendritic blocks. To test this, an 80 U/mL solution of trypsin was added to the Nile red-encapsulated micelles of PNVP-*b-dendr*(Phe-Lys)_2_ and PNVP-*b-dendr*(Phe-Lys)_3_. It was observed that time-dependence decreased in fluorescence intensity, indicating that the Nile red molecules were released from the hydrophobic cores of the micelles as they disassembled upon trypsin treatment (Figure 5). Nile red micelles without enzyme were used as a control under the same test conditions, where we found only a small amount of Nile red was released. In addition, the disassembly of Nile red was depended on the generation of dendrons in LDBCs. The higher the generation, the slower the disassembly rate (Figure 5c). When the length of hydrophilic linear chain PNVP was the same, the third-generation LDBC with more the number of hydrophobic Phe in its dendron and peripheral Fmoc groups had a slower enzymatic hydrolysis rate than the second-generation LDBC. Because the enzyme is not able to penetrate the PNVP shell into the micellar core to degrade the dendritic block, conversely, enzymatic degradation occurs at a “monomeric” form of amphiphilic LDBC, which is in equilibrium with the micelle aggregate [11] (Figure 6).

**Figure 7:**
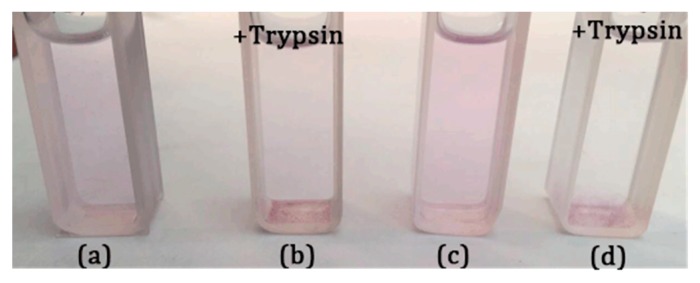
PNVP-*b*-*dendr*(Phe-Lys)_2_ (**a**,**b**) and PNVP-*b*-*dendr*(Phe-Lys)_3_ (**c**,**d**) loaded with Nile red and loaded with Nile red after the incubation 48 h with trypsin.

Particle sizing measurement was used to confirm the enzyme triggered disassembly of the LDBC micelles. Figure 3 shows that the particle size of copolymers micelles loaded with Nile red dramatically increased, from below 270 nm to more than 4000 nm, upon the addition of an 80 U/mL solution of trypsin for 48 h. This is in good agreement with the enzyme responsive feature observed with the naked eye (Figure 7). The encapsulation of Nile red by micelles made the micellar solution appear pink. After adding trypsin for 48 h, the color of the solution gradually became lighter and generated precipitates. The producing precipitate of PNVP-*b*-*dendr*(Phe-Lys)_3_ after the incubation 48 h with trypsin was isolated through centrifugation and subsequently analyzed by ^1^H NMR spectroscopy. Compared with ^1^H NMR spectrum of PNVP-*b*-*dendr*(Phe-Lys)_3_, the resonance signals of the protons of PNVP in ^1^H NMR spectrum of the precipitate almost disappeared, which demonstrated the precipitate consists mainly of (Fmoc)Lys(Fmoc)–OH and Phe–OH (Figure S5). In order to further examine if the disassembly occurs only due to the enzymatic hydrolysis of dendritic Phe-Lys, a solution of the same trypsin concentration was added to PNVP–NH_2_, the linear chain of the LDBCs, where we do not observe any precipitates. It suggests the enzyme-specific disassembly of the LDBC micelles. The enzyme-induced disassembly of the LDBC micelles was also supported by TEM studied. As shown in Figure 4c,f, we observed unstructured and large aggregates instead of spherical structures in the aqueous solution of Nile red-loaded micelles after treatment with trypsin.

### 3.4. In Vitro Drug Release from DOX-Loaded Micelles

**Figure 8:**
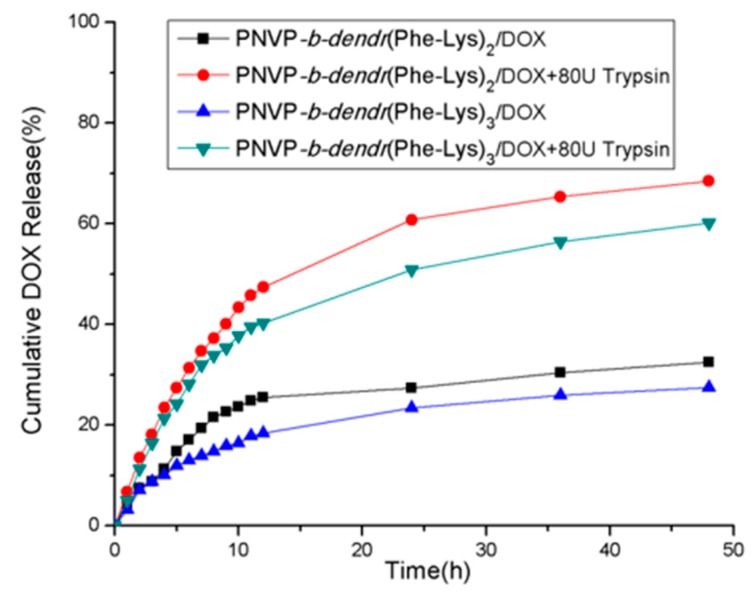
Release profile of drug-loaded micelles over time after addition of trypsin.

We investigated the trypsin-triggered release of doxorubicin (DOX) from the LBDCs micelles in PBS buffer (pH = 7.4) at 37 °C to simulate the physiological environment. The encapsulation efficiency (EE) of DOX in PNVP-*b*-*dendr*(Phe-Lys)_2_ and PNVP-*b*-*dendr*(Phe-Lys)_3_ micelles was 71.2% and 77.6%, respectively, when the feeding amount of DOX/ LBDCs (wt/wt) was <5%. The release of DOX in micelles was very low about 30% at 48 h in the absence of trypsin. However, DOX-loaded micelles showed significant release of DOX with about 15% release at 2 h and about 65% release at 48 h after the addition of an 80 U/mL solution of trypsin (Figure 8). The cumulative release of DOX-loaded micelles was not very accurate because the copolymers produced insoluble precipitate upon enzymatic activation and was mixed with DOX. The mixture was stirred in PBS buffer (pH = 5) to fully dissolve the DOX and then the fluorescence intensity of the resulting solution was measured. We found that the cumulative release of the drug reached more than 80% at 48 h upon trypsin treatment. The reason for the remarkably increase of DOX release may arise from the enzyme-induced disassembly of DOX-loaded micelles at physiological pH condition.

### 3.5. In Vitro Cytotoxicity of the LDBCs

**Figure 9:**
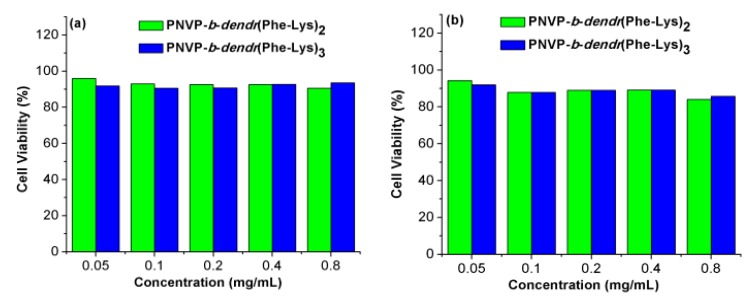
Cell viability of BEAS-2B (**a**) and SMMC-7721 (**b**) cells versus PNVP-*b*-*dendr*(Phe-Lys)_2_ and PNVP-*b*-*dendr*(Phe-Lys)_3_.

To apply the LDBCs in drug delivery field, the biocompatibility of PNVP-*b*-*dendr*(Phe-Lys)_2_ and PNVP-*b*-*dendr*(Phe-Lys)_3_ was investigated by cytotoxicity assessments. The LDBCs at varying concentrations were incubated with human lung epithelial cells (BEAS-2B) or human liver cancer cells (SMMC-7721) for 48 hours. The viability of BEAS-2B and SMMC-7721 cells after incubation with the copolymers was determined via MTS assay. As shown in Figure 9, no apparent toxicity effect was found on BEAS-2B and SMMC-7721 by the LDBCs when the concentration of the LDBCs increased from 0.05 to 0.8 mg/mL. These results demonstrate that PNVP-*b*-*dendr*(Phe-Lys)_2_ and PNVP-*b*-*dendr*(Phe-Lys)*_3_* have a good biocompatibility within limit concentration and are suitable for drug delivery.

## 4. Conclusions

In summary, we reported the design and synthesis of a novel class of enzyme responsive amphiphilic LDBCs (PNVP-*b*-*dendr*(Phe-Lys)_n_, n = 1–3) consisted of a hydrophilic linear PNVP and hydrophobic dendritic Phe-Lys dipeptides. The amphiphiles were synthesized through a combination of RAFT/MADIX polymerization of *N*-vinylpyrrolidone and by stepwise condensation approach of peptide chemistry. Specifically, we have demonstrated the second and the third generation LDBCs self-assemble to form spherical micelles under aqueous condition. The micelles were able to disassemble and release the encapsulated anticancer drug doxorubicin or hydrophobic dye Nile red upon trypsin treatment. Unlike classical amino acid-based LDBCs which contain just one kind of amino acid such as L-lysine, the dipeptide Phe-Lys are used as branch units, which could not only increase hydrophobicity which is essential for the formation of stable micelles but also be cleaved by protease trypsin. The disassembly of the micelles and release rates of the drug or dye can be adjusted by changing the generation of dendrons in PNVP-*b*-*dendr*(Phe-Lys)_n_. The higher the generation, the more stable the micelles and the slower the disassembly rate. MTS assays indicate that the LDBCs are well tolerated by different cell lines. This design and simple synthetic method will hopefully stimulate the manufacture of more enzyme-responsive polymer micelles in the future field of drug delivery.

## Figures and Tables

**Table 1:** Characterization of copolymers and their DOX-loaded micelles.

Sample	*M*_n,th_^a^ (kDa)	Weight Ratio of Hydrophobic Dendron ^b^ (%)	*M*_n,NMR_ (kDa) (DP) ^c^	*M*_n,GPC_^d^ (kDa)	Ð ^d^ (*M*_w_/*M*_n_)	DL ^e^ (w/w, %)	EE ^e^ (w %)
PNVP	9.8	-	10.0 (88)	29.0	1.28	-	-
PNVP-*b*-*dendr*(Phe-Lys)	10.6	8	-	33.8	1.34	-	-
PNVP-*b*-*dendr*(Phe-Lys)_2_	11.6	16	-	35.4	1.32	2.7	71.2
PNVP-*b*-*dendr*(Phe-Lys)_3_	13.6	28	-	37.6	1.32	3.0	71.6

^a^ The theoretic molecular weight was calculated by the formula: *M*_n,th_ = Mmonomer × ((monomer)/(CTA-Boc)) × Conversion% + MCTA-Boc. ^b^ Weight ratios based on theoretical values. ^c^ The degree of polymerization of the copolymers was calculated using *M*_n,NMR_. ^d^ *M*_n__,GPC_ and PDI were determined by GPC in relative to PMMA standards. ^e^ Drug loading (DL) and encapsulation efficiency (EE) of DOX-loaded micelles was calculated by the formulae in Experimental Section.

## Supplementary Materials

The following are available online at https://www.mdpi.com/2073-4360/11/10/1625/s1, Figure S1: ^1^H NMR spectra of PNVP-*b*-*dendr*(Phe-Lys)_2_ in DMSO-d_6_ (a) and D_2_O (b), Figure S2: ^1^H NMR spectra of PNVP-*b*-*dendr*(Phe-Lys)_3_ in DMSO-d_6_ (a) and D_2_O (b), Figure S3: Fluorescence spectra of Nile red in phosphate buffer solution (pH 7.4) at different concentrations (a) and ploted of maximum emission intensity versus logarithm of concentration (b) of PNVP-*b*-*dendr*(Phe-Lys)_2_, Figure S4: Fluorescence spectra of Nile red in phosphate buffer solution (pH 7.4) at different concentrations (a) and ploted of maximum emission intensity versus logarithm of concentration (b) of PNVP-*b*-*dendr*(Phe-Lys)_3_, Figure S5: ^1^H NMR spectra in DMSO-d_6_ for (Fmoc)Lys(Fmoc)–OH (a), the precipitation of PNVP-*b*-*dendr*(Phe-Lys)_3_ after the incubation 48 h with trypsin (b) and PNVP-*b*-*dendr*(Phe-Lys)_3_ (c).
