# Supplementary material for: Self-Assembly and Enzyme Responsiveness of Amphiphilic Linear-Dendritic Block Copolymers Based on Poly(N-vinylpyrrolidone) and Dendritic Phenylalanyl-lysine Dipeptides
Source: Polymers (Basel). 2019 Oct 8;11(10):1625. doi: 10.3390/polym11101625 (PMC6836210; doi:10.3390/polym11101625)
Supplement: Supplementary file 1 [file polymers-11-01625-s001.pdf]

polymers

Supporting Information

Self-assembly and Enzyme Responsiveness of Amphiphilic Linear-Dendritic Block Copolymers Based on Poly(*N*-vinylpyrrolidone) and Dendritic Phenylalanyl-lysine Dipeptides

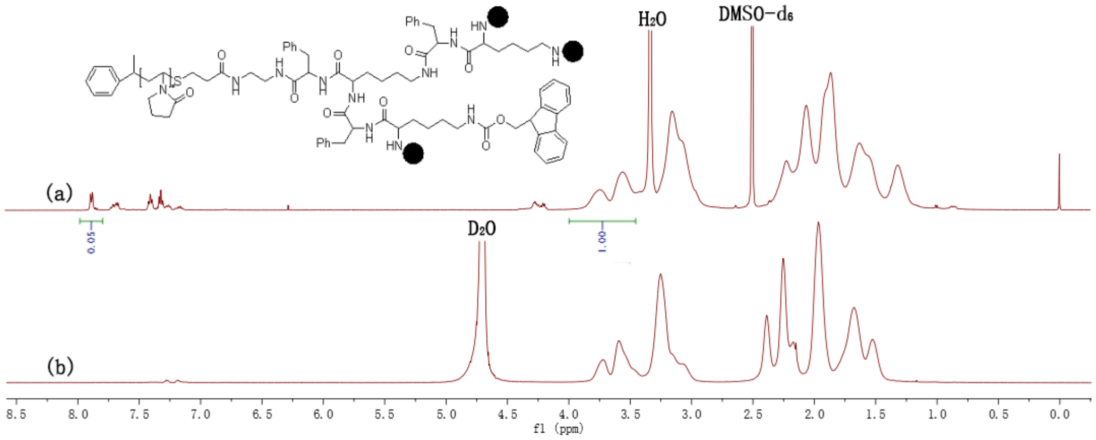

Figure S1. ^{1}H NMR spectra of PNVP-*b*-*dendr*(Phe-Lys)$_2$ in DMSO-d_6 (**a**) and D_2O (**b**).

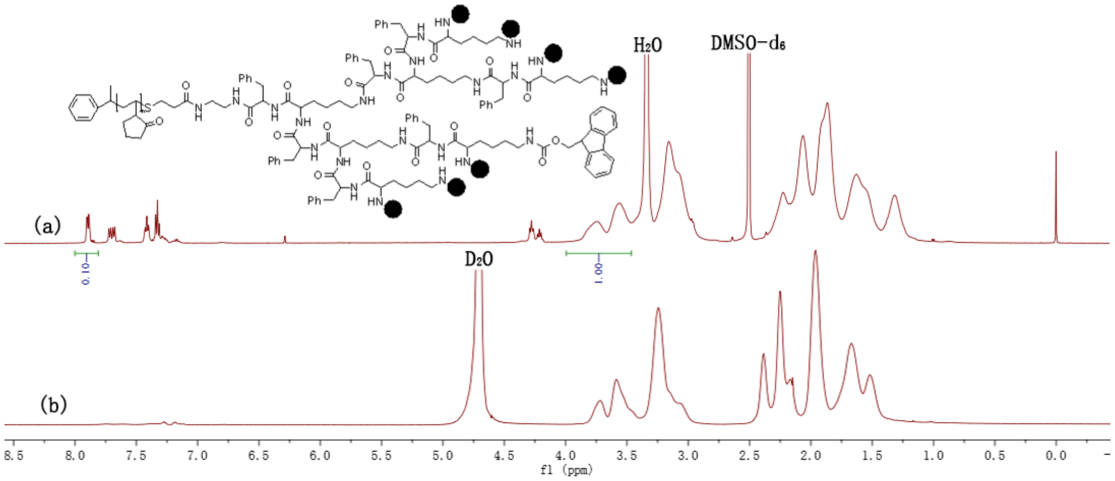

Figure S2. ^{1}H NMR spectra of PNVP-*b*-*dendr*(Phe-Lys)$_3$ in DMSO-d_6 (**a**) and D_2O (**b**).

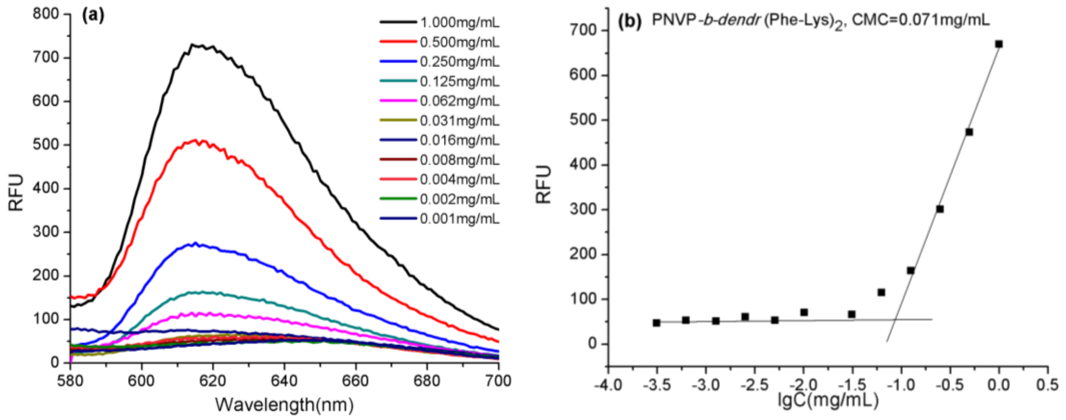

Figure S3. Fluorescence spectra of Nile red in phosphate buffer solution (pH 7.4) at different concentrations (**a**) and ploted of maximum emission intensity versus logarithm of concentration (**b**) of PNVP-*b*-*dendr*(Phe-Lys)$_2$.

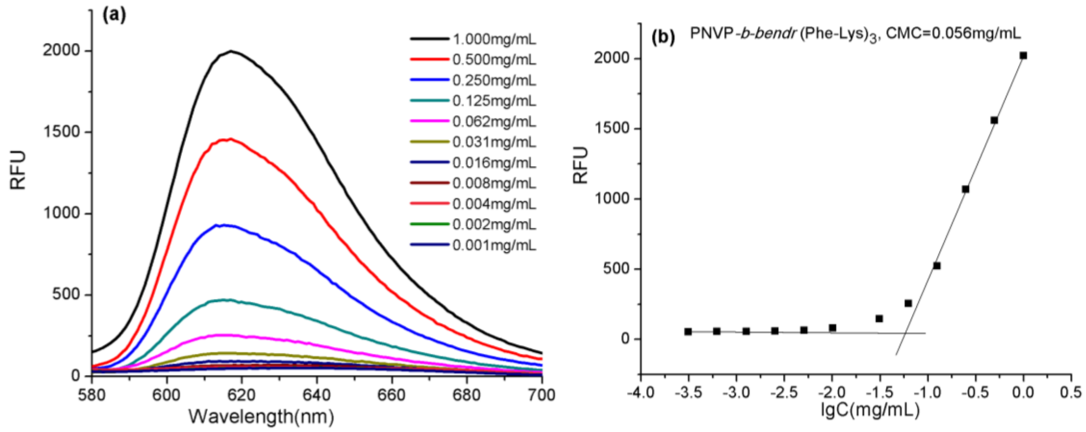

Figure S4. Fluorescence spectra of Nile red in phosphate buffer solution (pH 7.4) at different concentrations (**a**) and ploted of maximum emission intensity versus logarithm of concentration (**b**) of PNVP-*b*-*dendr*(Phe-Lys)$_3$.

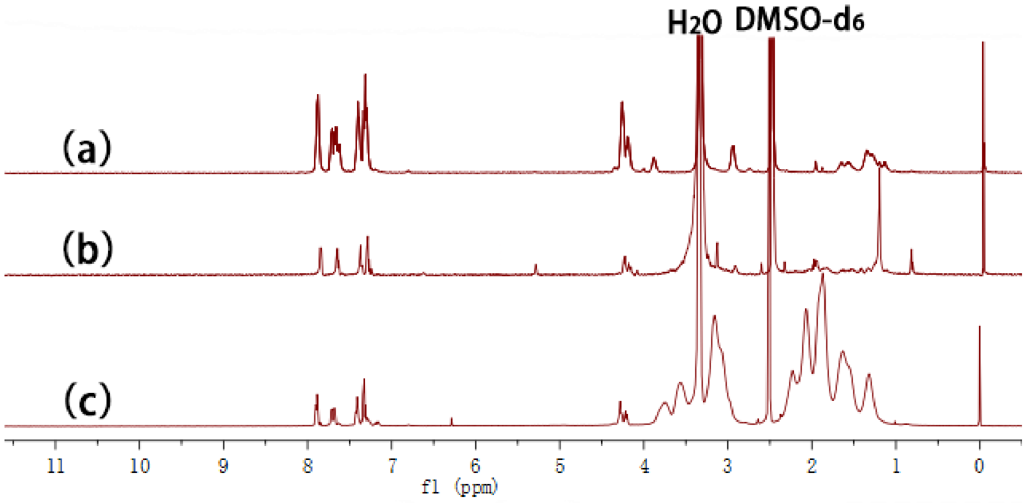

Figure S5. ^{1}H NMR spectra in DMSO-d$_6$ for (Fmoc)Lys(Fmoc)–OH (**a**), the precipitation of PNVP-*b*-*dendr*(Phe-Lys)$_3$ after the incubation 48 h with trypsin (**b**) and PNVP-*b*-*dendr*(Phe-Lys)$_3$ (**c**).
